# The protective effects of MSC‐EXO against pulmonary hypertension through regulating Wnt5a/BMP signalling pathway

**DOI:** 10.1111/jcmm.16002

**Published:** 2020-10-22

**Authors:** Zhaohua Zhang, LiLi Ge, Shanshan Zhang, Jue Wang, Wen Jiang, Qian Xin, Yun Luan

**Affiliations:** ^1^ Department of Pediatrics The Second Hospital Cheeloo College of Medicine Shandong University Jinan China; ^2^ The Second Hospital Cheeloo College of Medicine Shandong University Jinan China; ^3^ Department of Cardiac Ultrasound The Second Hospital Cheeloo College of Medicine Shandong University Jinan China; ^4^ Department of Emergency The Second Hospital Cheeloo College of Medicine Shandong University Jinan China; ^5^ Central Research Laboratory Institute of Medical Science The Second Hospital Cheeloo College of Medicine Shandong University Jinan China

**Keywords:** BMPR2, MSC‐EXO, PH, pulmonary vascular remodelling, Wnt5a

## Abstract

The aim of the study was to explore the mechanism of mesenchymal stem cell‐derived exosomes (MSC‐EXO) to protect against experimentally induced pulmonary hypertension (PH). Monocrotaline (MCT)‐induced rat model of PH was successfully established by a single intraperitoneal injection of 50 mg/kg MCT, 3 weeks later the animals were treated with MSC‐EXO via tail vein injection. Post‐operation, our results showed that MSC‐EXO could significantly reduce right ventricular systolic pressure (RVSP) and the right ventricular hypertrophy index, attenuate pulmonary vascular remodelling and lung fibrosis in vivo. In vitro experiment, the hypoxia models of pulmonary artery endothelial cell (PAEC) and pulmonary vascular smooth muscle cell (PASMC) were used. We found that the expression levels of Wnt5a, Wnt11, BMPR2, BMP4 and BMP9 were increased, but β‐catenin, cyclin D1 and TGF‐β1 were decreased in MSC‐EXO group as compared with MCT or hypoxia group in vivo or vitro. However, these increased could be blocked when cells were transfected with Wnt5a siRNA in vitro. Taken together, these results suggested that the mechanism of MSC‐EXO to prevent PH vascular remodelling may be via regulation of Wnt5a/BMP signalling pathway.

## 1 INTRODUCTION

Pulmonary arterial hypertension (PH) is a chronic disease that ultimately progresses to right‐sided heart failure and death, defined clinically as mean pulmonary arterial pressure > 25 mm Hg at rest with normal left atrial pressure,^1^ which is characterized by small pulmonary dysfunction and structural remodelling and right ventricular (RV) failure.^2^, ^3^ Abnormal pulmonary vascular cell proliferation plays a central role in the occurrence and development of PH.^4^, ^5^, ^6^ Currently, there were a lot of drugs used to improve the clinical symptoms of PH patients; however, these treatments cannot reverse the pulmonary vascular remodelling process and prevent PH development. Therefore, novel approaches are urgently needed.

The pathogenesis of PH is complicated, and the damage of pulmonary artery endothelial cell (PAEC) is a crucial early onset. Reports showed that the proliferation and angiogenesis of PAEC are underlying mechanisms for the pathological features of PH. Thus, revealing the potential molecular and cellular mechanism of endothelial cell angiogenesis underlies PH is very important and can help to explore new treatment strategies. Wnt5a is one of a member of the Wingless (Wnt) and activates non‐canonical or canonical Wnt signalling pathways through specific coupling of different receptor. Down‐regulation of Wnt5a could promote hypoxia‐induced pulmonary vascular smooth muscle cell (PASMC) proliferation,^7^ and loss of Wnt5a could reduce the formation of new vessels in PH.^1^ Thus, the production of Wnt5a is likely to become a new way to prevent the vessel loss in PH.

Cells isolated from Wharton's jelly, referred to as umbilical cord matrix stromal (UCMS) cells, express mesenchymal stromal cell (MSC) surface markers, self‐renew and are multipotent (differentiate into bone, fat, cartilage, etc.) in vitro. Human UCMSC lacks of an apparent host immune response,^8^ human UCMS‐derived exosomes (MSC‐EXO) is one of the main therapeutic vesicles, work from our group confirmed that intravenous delivery of MSC‐EXO could inhibit experimental PH vascular remodelling and right ventricular impairments.^9^, ^10^, ^11^ Although MSC‐EXO is a promising potential target for new therapies of PH, the mechanism was not very clear. The aim of the study was to explore the mechanism of MSC‐EXO to protect against experimentally induced PH in vivo and vitro.

## 2 MATERIALS AND METHODS

### 2.1 Animal model

Male Wistar rats weighing 200 g to 250 g were purchased from animal centre of Second Hospital of Shandong University. Male rats were used to minimize hormonal effects (eg of oestrogen).^12^, ^13^, ^14^ The animal protocols followed the guidelines of the Institutional Animal Care and Use Committee (IACUC) of Shandong University. All rats received humane care in compliance with the Guide for the Care and Use of Laboratory Animals published by the US National Institute of Health.

### 2.2 Preparation of exosomes

Isolation of MSCs from human umbilical cord Wharton’s Jelly with as previously described with some modifications.^15^ The immunotyping characterization of hUCMSCs occurred at passage 3‐4 using human‐specific antibodies CD34, CD45, CD73, CD90, CD105 and HLA‐DR (BD Biosciences Pharmingen, San Diego, CA) by a fluorescence‐activated cell sorter (FACS, BD FACSAria II). Cell differentiation ability was performed by differentiation media and supplements (Cyagen US Inc).

When reached 90% confluences, the adherent cells were incubated in medium with 5% exosome‐depleted FBS (ExoDP‐FBS, SBI‐ EXO‐FBS‐50A‐1) for 24 hours, and 5‐8 passage hUCMSC were used for experiments. The conditioned medium was centrifuged at 4°C at 300 *g* for 10 minutes at 2000 *g* for 10 minutes and finally at 10 000 *g* for 30 minutes to remove the cells and debris, followed by centrifugation of the supernatant at 100 000 *g* at 4°C for 1 hour. MSC‐EXO were resuspended in PBS and filtered with a 0.22 µm microfiltration membrane, centrifuged again in PBS at 100 000 *g* for 1 hour to collect the exosomes. The protein concentration of hUCMSC‐EXO was determined using a bicinchoninic acid (BCA) assay kit. Transmission electron microscope (TEM) was used to detect the morphology of MSC‐EXO according to the manufacturer’s instructions. Briefly, the prepared exosomes were stained with phosphotungstic acid solution and then performed under a Hitachi‐9000 TEM system. The exosome markers CD63, CD81, TSG101 and ALIX were analysed by Western blot. β‐actin expression was used as an internal control.

### 2.3 Experimental design

We established rat PH model through a single intraperitoneal injection of MCT (50 mg/kg; Sigma, St. Louis, MO, USA). 25 µg of MSC‐EXO in 200 µL PBS via tail vein injection form 21 to 23 days.^15^ Four weeks later, there are 3 rats in MCT group and 2 rats in MSC‐EXO group deaths. The surviving experimental animals were divided into 3 groups (n = 10, 7, and 8): Control (saline‐treated) group, MCT group, and MSC‐EXO group. Four weeks later, the rats were anesthetized with pentobarbital (30 mg/kg, ip, Sigma‐Aldrich) and inserted with a 3F‐Miller micro‐tip catheter via the right jugular vein into the right ventricle (RV) to obtain the heart rate (HR), cardiac output (OC) and right ventricular systolic pressure (RVSP).

### 2.4 Histology and immunology

Post‐operation, the heart was obtained quickly, and the weight ratio of the right ventricular (RV) to left ventricle (LV) plus the septum (LV+S) and the right ventricular weight was calculated to quantify the right ventricular hypertrophy. The lung tissues were fixed and embedded in paraffin, and the serially sections at a thickness of 4‐5 µm were stained with haematoxylin‐eosin stain. The vascular wall thickness (WT), vascular external diameter (ED), vascular wall area (WA) and total vascular area (TA) to calculate WT% (WT/ED) and WA% (WA/TA) were measured as previously study. On the other hand, the serially sections were stained with Masson’s trichrome to measure the evaluation of the degree of fibrosis. The average of the 10 high‐power fields (hpf) was randomly selected, and positively stained areas were padded with a single colour and converted into pixels through optical density (OD) calibration.

Immunohistochemistry was used to analyse the expression of CD31; on the other hand, we detected the expression of Histone H3 (p‐H3) in pulmonary artery smooth muscle cells by double fluorescent staining of p‐H3 and α‐smooth muscle actin (SMA) in lung section. Briefly, after blocking with 5% bovine serum albumin for 30 minutes at room temperature, the lung sections were incubated overnight at 4°C with anti‐CD31 (AF3628), α‐SMA (ab21027), V‐E cadherin (CST#14472) and Histone H3 antibodies (p‐H3, ab14955). Then, sections were further incubated with a second antibody for 2 hours at room temperature. Subsequently, the 3, 3′‐ diaminobenzidine (DAB) dye was added to visualize the antibodies. For immunofluorescence, the sections were followed by 1‐h incubation in the dark with florescence isothiocyanate‐conjugated secondary antibody. Images were taken with ZEISS LSM800 confocal microscope (Tokyo, Japan). All experiments were performed by two examiners blinded to treatment assignment.

### 2.5 Cell culture and siRNA preparation

Healthy rat PAEC and PASMC were purchased from Procell Life Science&Technology Co, Ltd. (Wuhan, China) and cultured in special culture medium (Procell, China) supplemented with 100 Ug/mL of penicillin, 100 IU/mL streptomycin and 10% (vol/vol) foetal bovine serum (FBS) at 37°C in a humidified normoxia condition (21% O_2_, 5% CO_2_, 74% N_2_) or a hypoxic cell incubator in hypoxia condition (3% O_2_, 5% CO_2_, 92% N_2_). At different time points, cells were treated with MSC‐EXO fraction (100 μg/mL), respectively.

Here, the gene of Wnt5a was knocked down in PAEC and PAMSC by transfecting siRNA targeting. The siRNA oligonucleotides were selected to correspond to the nucleotide sequence of si‐r‐Wnt5a: 5′‐GGACAACACTTCTGTCTTT‐3′. Total RNA was extracted from cells using a Qiagen RNeasy kit (Qiagen, Basel, Switzerland). Complementary DNA (cDNA) first strand was produced using a Superscript first‐strand synthesis system using oligo (dt) antisense primers (Invitrogen, Lucerne, Switzerland). Amplified fragments were analysed in 1.5% agarose gel electrophoresis in the presence of ethidium bromide (Sigma‐Aldrich). GAPDH was used as an internal control for the amount of RNA input.

### 2.6 Cell proliferation assays and BrdU incorporation

Cell proliferation was monitored using a cell counting kit (CCK)‐8 assay, Briefly, PAEC were seeded into 96‐well plates at about at a density of 5 × 10^3^ cells/well. The cells were subjected to hypoxia with or without MSC‐EXO at different time points, and then, CCK‐8 reagent (10 µL) was added to each well and further incubated for 3 hours. The absorbance was measured at 450 nm in a spectrophotometer.

The Bromodeoxyuridine (BrdU) incorporation assays were implemented according to the manufacturer’s instructions. Briefly, cultured PAEC and PASMC in 96‐well culture plates were incubated with 5‐BrdU labelling solution for approximately 48 hours, anti‐BrdU monoclonal antibody was incubated for 1 hour, and goat anti‐mouse IgG was conjugated. The absorbance of the sample was detected using a spectrophotometer microplate reader at 450/550 nm.

### 2.7 Real‐time PCR and Western blot

Quantitative real‐time polymerase chain reaction (qRT‐PCR) analysis was performed to detect the relative expression of CD31, a‐SMA and V‐E cadherin using gene‐specific primers as described previously. Briefly, total RNA in lung tissues or PAEC was extracted using RNeasy kit (Qiagen, Valencia, CA). ABI Prism 7900 sequence detection system software (version 2.2) was used to analyse the data, and GAPDH was used as an internal control for input RNA. The primers were designed by the Primer Express software package.

The protein expression were detected by Western blot; briefly, antibodies of CD63 (Invitrogen, 10628D), CD81 (MA5‐32333), TSG101 (MA5‐32463), CD31 (AF3628), α‐SMA (ab21027), V‐E cadherin (CST#14472), BMPR2 (ab130206), Wnt5a (ab174963), Wnt11 (ab31962), β‐catenin (ab32572), cyclin D1 (MA5‐15512) and PCNA (ab92552), BMP4 (ab39973), BMP9 (ab35088), transforming growth factor‐β1 (TGF‐β1;ab25121), Smad2/3 (sc‐133098), p‐Smad2/3 (sc‐11769), Smad1/5/8 (sc‐6031) and p‐Smad1/5/8 (sc‐12353) were used respectively, overnight at 4°C. The primary antibody‐labelled membranes were then treated with the horseradish peroxidase (HRP)‐conjugated goat anti‐rabbit secondary antibody to IgG (ab205718) at room temperature for 1.5 hours. GAPDH expression was used as an internal control.

### 2.8 Cell invasion and migration assays

A 5 × 10^5^ PAECs were plated onto 24‐well plates and grown at 37 °C/5% CO_2_ for 48 hours to a confluence of >90%. The medium was removed, and in the cell monolayers, a wound line was created by manually scraping the cells with a 10 µL plastic pipette tip. Debris was removed from the culture by washing with PBS two times. When the cells were stably transfected with Wnt5a and treated with or without 100ng/ml MSC‐EXO, then they were incubated for another 12 hours. Images were captured using Nikon Eclipse 90i microscope (magnification, ×100).

The migratory function of PAEC was measured by a modified Boyden chamber (Transwell; Corning Life Sciences, Inc., Tewksbury, MA, USA). In brief, cells in each group were treated with complete medium containing 1% FBS and were added into the upper chamber with 8.0 μm pore polycarbonate membrane insert pre‐coated with 30 μL of reduced‐growth factor Matrigel (Corning Inc., Tewksbury, MA, USA), Matrigel was thawed at 4°C overnight. 48‐hour later, the cells that had not migrated was wiped off the upper side of the filter using a cotton swab. Migrated cells were fixed with 4% paraformaldehyde/PBS for 10 minutes and subsequently stained with crystal violet for 1 hour. Images were captured using a Nikon Eclipse inverted optical microscope, and the number of migrated cells was counted. These experiments were repeated three times.

### 2.9 Statistical analysis

Data of continuous variables are presented as mean ± standard deviation (SD), while data not conforming to homogeneity of variance or normal distribution were expressed as interquartile range. Comparisons of mean values between two groups were analysed using a non‐paired t test. Comparisons among multiple groups were analysed by one‐way analysis of variance (ANOVA), followed by post hoc test. Statistical analysis was carried out by using the SPSS 1.9 software (IBM, Armonk, NY, USA). *P* < 0.05 was regarded as significant statistical difference.

## 3 RESULTS

### 3.1 Characterization and differentiation potential of hUCMSCs

The MSC surface markers were determined by fluorescence‐activated cell sorting (FACS) showed that the majority of hUCMSCs expressed high levels of the CD73, CD90 and CD105 markers, whereas CD34, CD45 and HLA‐DR markers were relatively absent (Figure S1A). On the other hand, according to the Alizarin Red‐S, Oil Red‐O and Toluidine blue staining, the cells had the ability to differentiate into osteocytes, adipocytes and cartilage (Figure S1B).

Characterization of the shape and size of MSC‐EXO derived from human umbilical cord was carried out by transmission electron microscope (Figure S1C); the range of MSC‐EXO was between 50 and 150 nm in size. The protein expression of MSC‐EXO markers was detected by Western blot, as shown in Figure S1D, an enrichment of CD63, CD81, ALIX and TSG101 levels in MSC‐EXO than MSC‐CM. In order to identify whether the MSC‐EXO released by MSCs could transfer to cells, the exosomes were traced by PKH26 and co‐cultured with PAMSC, which was observed under a confocal fluorescence microscopy at 0 h and 24 h after co‐culture (Figure S1E).

### 3.2 Effect of MSC‐EXO on pulmonary vascular remodelling

**Figure 1:**
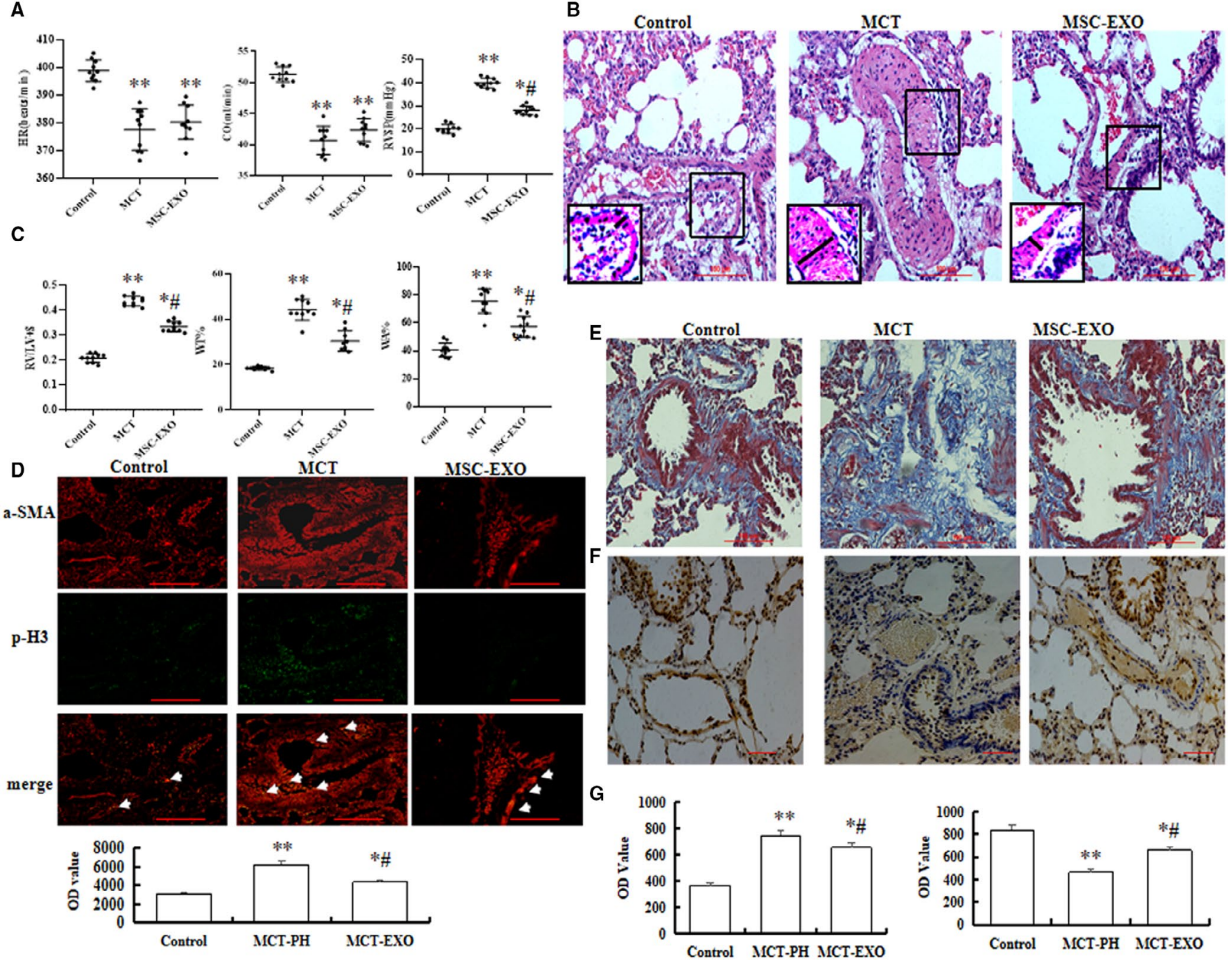
Effect of MSC‐EXO on MCT‐induced pulmonary artery pressure and vascular remodelling. A, Comparative analysis of heart rate (HR), cardiac output (OC) and right ventricular systolic pressure (RVSP). B, Representative haematoxylin and eosin staining images in each group. C, Comparative analysis of the right ventricular (RV) to left ventricle (LV) plus the septum (LV+S) ratios [RV/(LV+S)] and the per cent of wall thickness and wall area. D, Double immunofluorescence staining analysis of a‐SMA(red) and p‐H3 (green) in lung tissue. E, Masson's staining, F, Vessel analysis by immunohistochemistry using anti‐CD31 antibodies in lung. G, Comparative analysis of optical density (OD) value to detection the fibrosis and the positive staining of CD3 in lung tissue. *P < 0.05 vs control group; ^#^ *P* < 0.05 vs MCT group. Red bar = 100 µm

**Figure 2:**
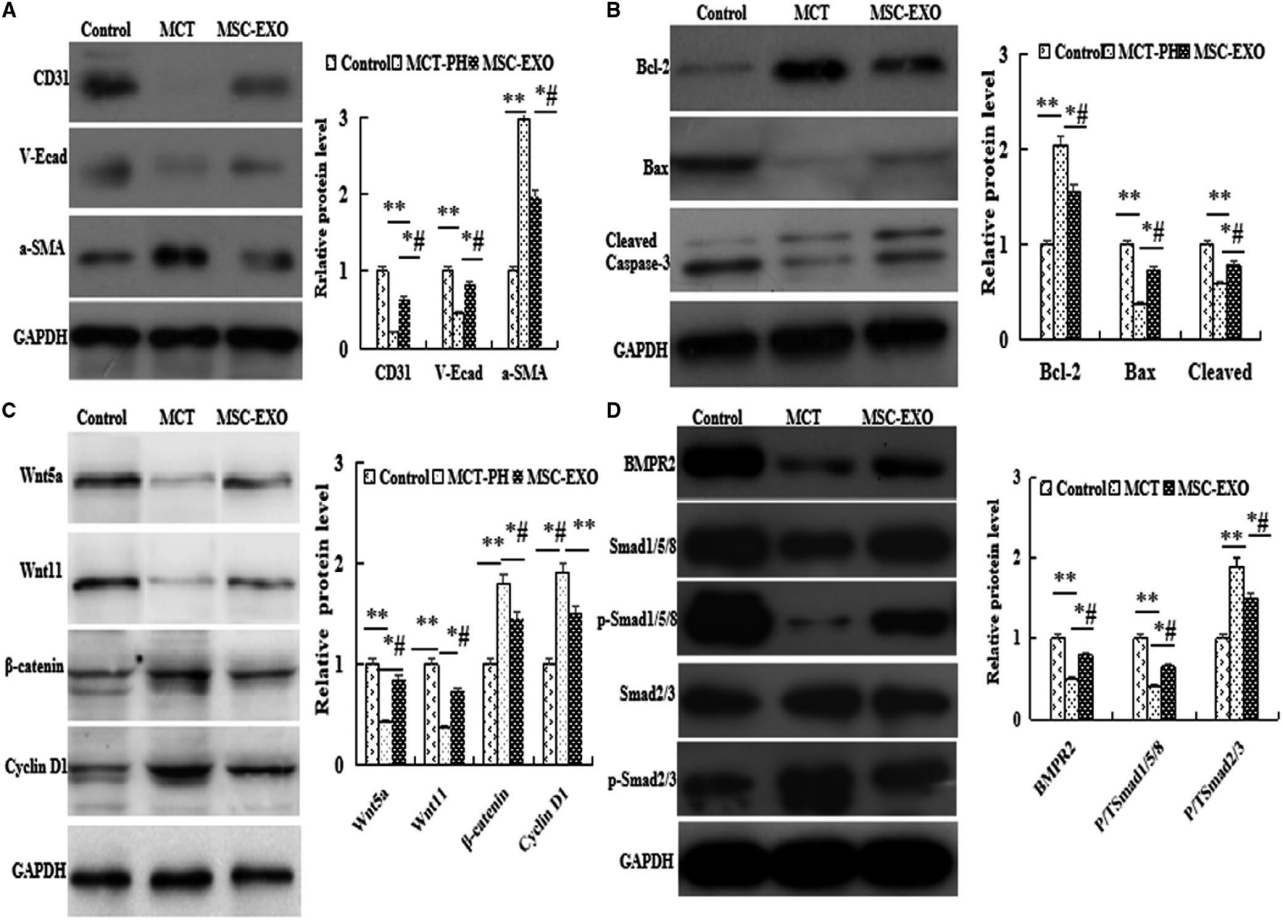
The protein expression analysis by Western blot in lung tissue. A, a‐SMA, CD31 and V‐E cadherin; B, Bcl2, Bax and cleaved caspase‐3; C, Wnt5a, Wnt11, β‐catenin and cyclin D1; D, BMPR2, Smad1/5/8 and Smad2/3. *P < 0.05 vs control group; ^#^ *P* < 0.05 vs MCT group

Four weeks after MCT injection, our results showed that there was no significantly differ in HR and CO in MCT‐induced PH and MSC‐EXO rats (*P *> 0.05); however, a significant decrease of RVSP and RV/(LV + S) in MSC‐EXO administration rats than that in MCT‐induced PH rats (*P* < 0.05; Figure 1A). Moreover, WT% and MA% of muscular arteries were significantly increased in MCT group than in control, but notably decreased in MSC‐EXO group (*P* < 0.05; Figure 1B,C). The immunofluorescence results showed that positive expression of p‐H3 in MSC‐EXO was significantly lower in MSC‐EXO treatment group than that in MCT group (Figure 1D). Masson’s trichrome staining showed that there was obvious collagen deposition in the pulmonary interstitium in the MCT rats, but a significantly alleviated in MSC‐EXO treatment rats (Figure 1E,G *P* < 0.05), Immunohistochemical and Western blot results showed that the protein expression of CD31 and V‐E cadherin were significantly increased, respectively, but significantly decreased α‐SMA level in MSC‐EXO treatment group than that in MCT group (Figure 1F,G and Figure 2A *P* < 0.05).

### 3.3 Effect of MSC‐EXO on cell apoptosis and Wnt/BMP signalling pathway

In the present study, MCT‐induced cell apoptosis was observed by analysing the protein expression levels of antiapoptotic gene Bcl2, the proapoptotic gene caspase‐3 and Bax. The results showed that as compared with MCT‐PH group, the expression of Bcl2 was significantly decreased, but caspase‐3 and Bax were significantly increased in MSC‐EXO group (*P* < 0.05, Figure 2B).

Moreover, the protein expression of Wnt5a signalling pathway factors Wnt5a, Wnt11 β‐catenin and cyclin D1 were detected by Western blot, the results showed that the levels of Wnt5a and Wnt11 were significantly decreased, but β‐catenin and cyclin D1 levels were increased in MCT‐PH group than that in the control; however, these results were significantly reverse in MSC‐EXO treatment group (*P* < 0.05, Figure 2C). These results showed that MSC‐EXO attenuated PH pulmonary vascular remodelling through regulation of Wnt5/β‐catenin signal pathway.

Furthermore, the expression of BMP signalling axis molecules were determined, the results indicated that the protein expression of BMPR2 and the ratio of the ratio of pSmad to total Smad1/5/8 was higher, but pSmad to total Smad 2/3 was lower in MSC‐EXO group than that in MCT‐PH group (*P* < 0.05, Figure 2D). These results indicated that MSC‐EXO inhibited MCT‐PH pulmonary vascular remodelling also through regulation of BMP signalling pathways.

### 3.4 Effect of MSC‐EXO on cell proliferation, invasion and migration in vitro

**Figure 3:**
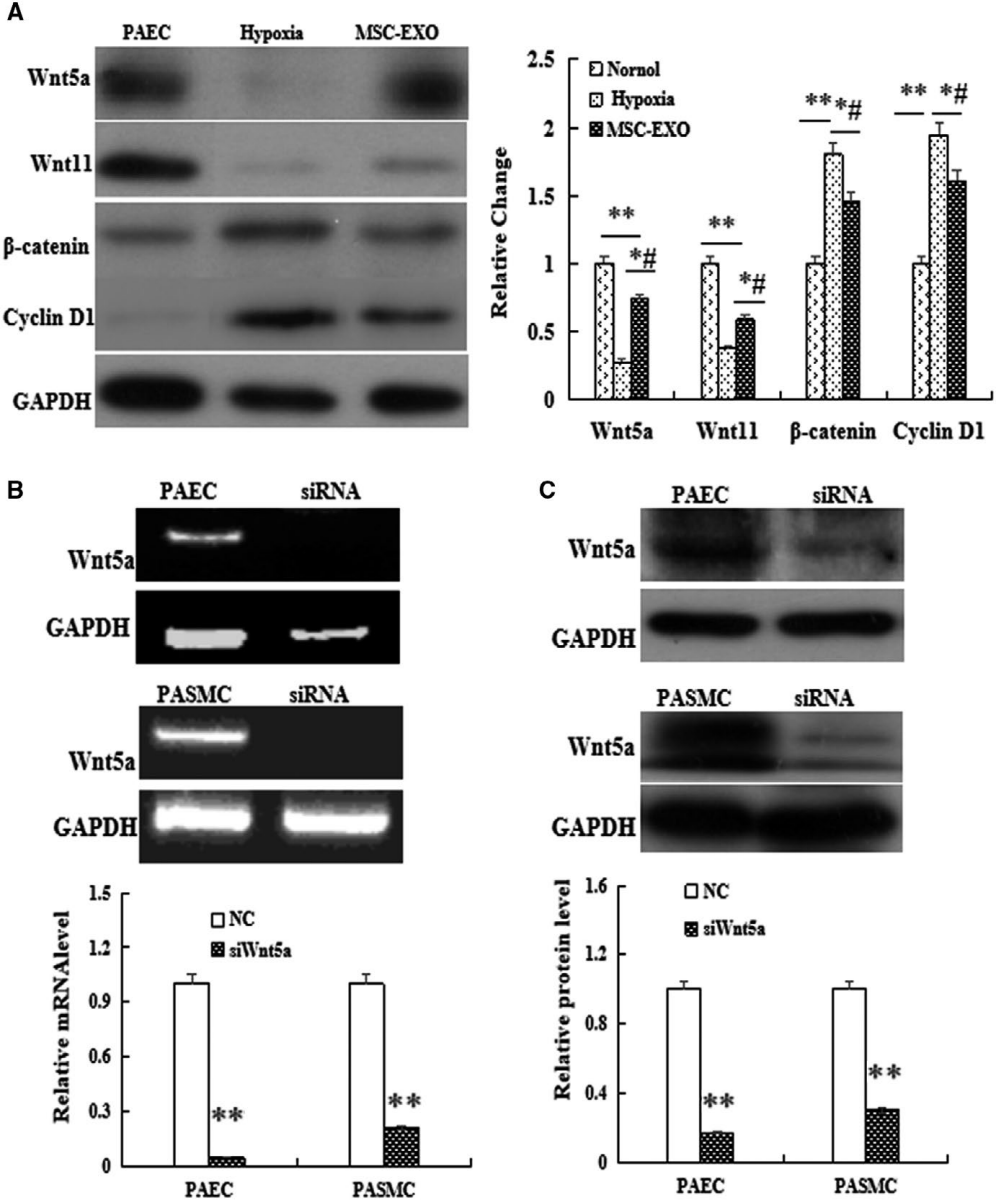
Effect of MSC‐EXO on hypoxia‐PAEC in vitro. A, the protein expression analysis of Wnt5a, Wnt11, β‐catenin and cyclin D1 by Western blot. B, Detection of Wnt5a mRNA expression in PAEC and PASMC by PCR. C, Detection of Wnt5a protein expression in PAEC and PASMC by Western blot. **P < 0.01 vs Normal group

The protein expression of Wnt5a signalling pathway factors was analysed by Western blot after the cells were hypoxia exposure for 72 hour. The results showed that the expression of Wnt5a and Wnt11 was significantly decreased, but β‐catenin and cyclin D1 were increased in hypoxia‐induced PAEC; however, these results were restored in MSC‐EXO treatment group as compared with hypoxia group (*P* < 0.05, Figure 3A). To investigate the role of Wnt5a pathway in MSC‐EXO, the gene of Wnt5a was knocked down by transfecting Wnt5a siRNA. As shown in Figure 3B, transfection of Wnt5a siRNA caused a reduction in Wnt5a expression and the inhibition efficacy was about 97.4 % and 92.8% at mRNA level at mRNA level, 50.8% and 70.4% at protein level in PAEC and PAMSC, respectively. More importantly, the above results were significantly reversed in Wnt5a siRNA group (*P* < 0.05, Figure 3B,C).

**Figure 4:**
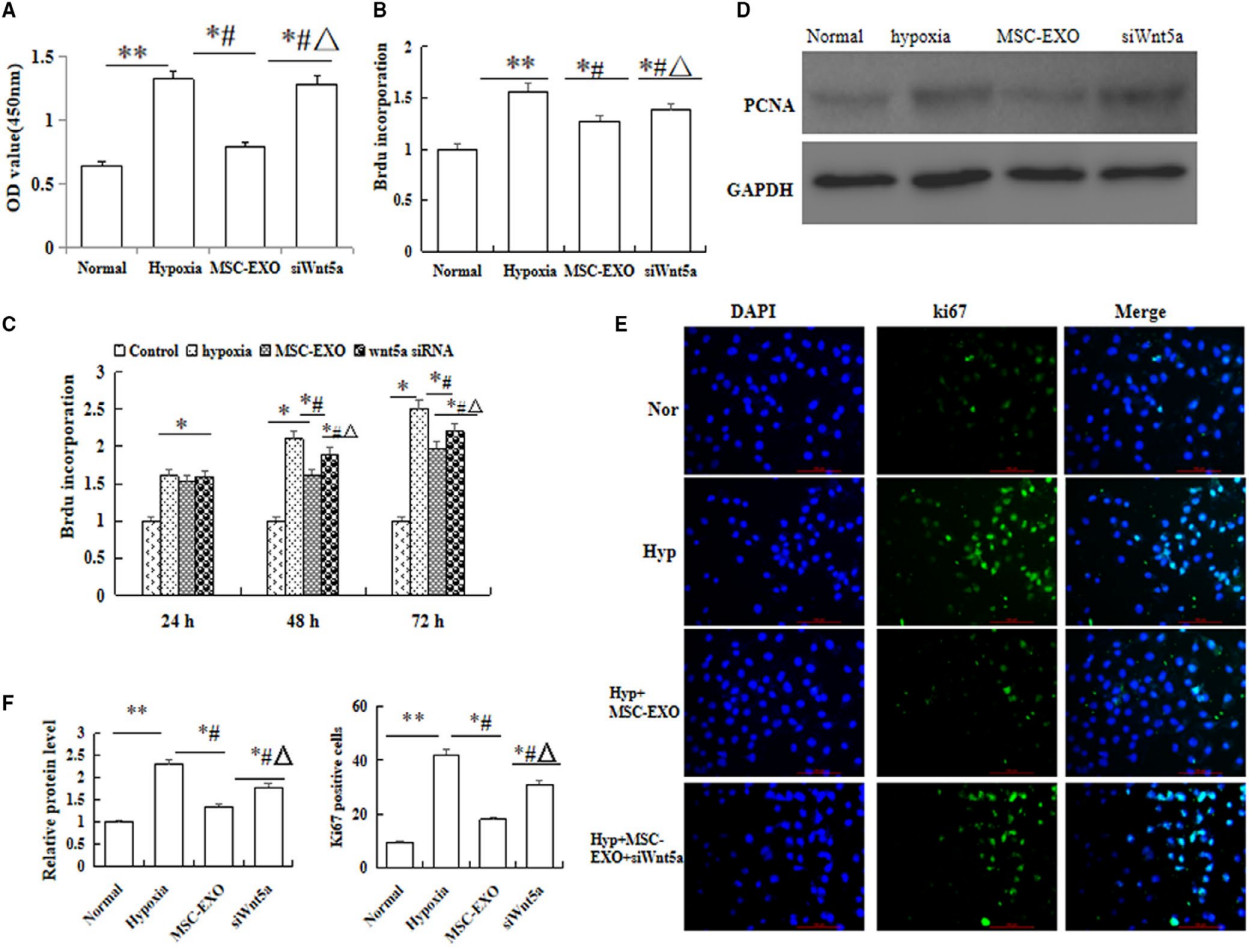
Effect of MSC‐EXO on hypoxia‐induced cell viability and proliferation. A, Cell counting kit (CCK)‐8 assay in PAEC. B, Proliferation analysis by BrdU cell proliferation assay in PAEC. C, Proliferation analysis by BrdU cell proliferation assay in PASMC in 24 h, 48 h and 72 h. D, Detection of proliferating cell nuclear antigen (PCNA) protein expression in PAEC by Western blot. E, Immunofluorescence staining with Ki67 antigen in PAEC. F, Comparative analysis of PCNA and ki67 protein expression. *P < 0.05 vs Normal group; ^#^ *P* < 0.05 vs hypoxia group; ^△^ *P *< 0.05 vs MSC‐EXO group. Red bar = 100 µm

**Figure 5:**
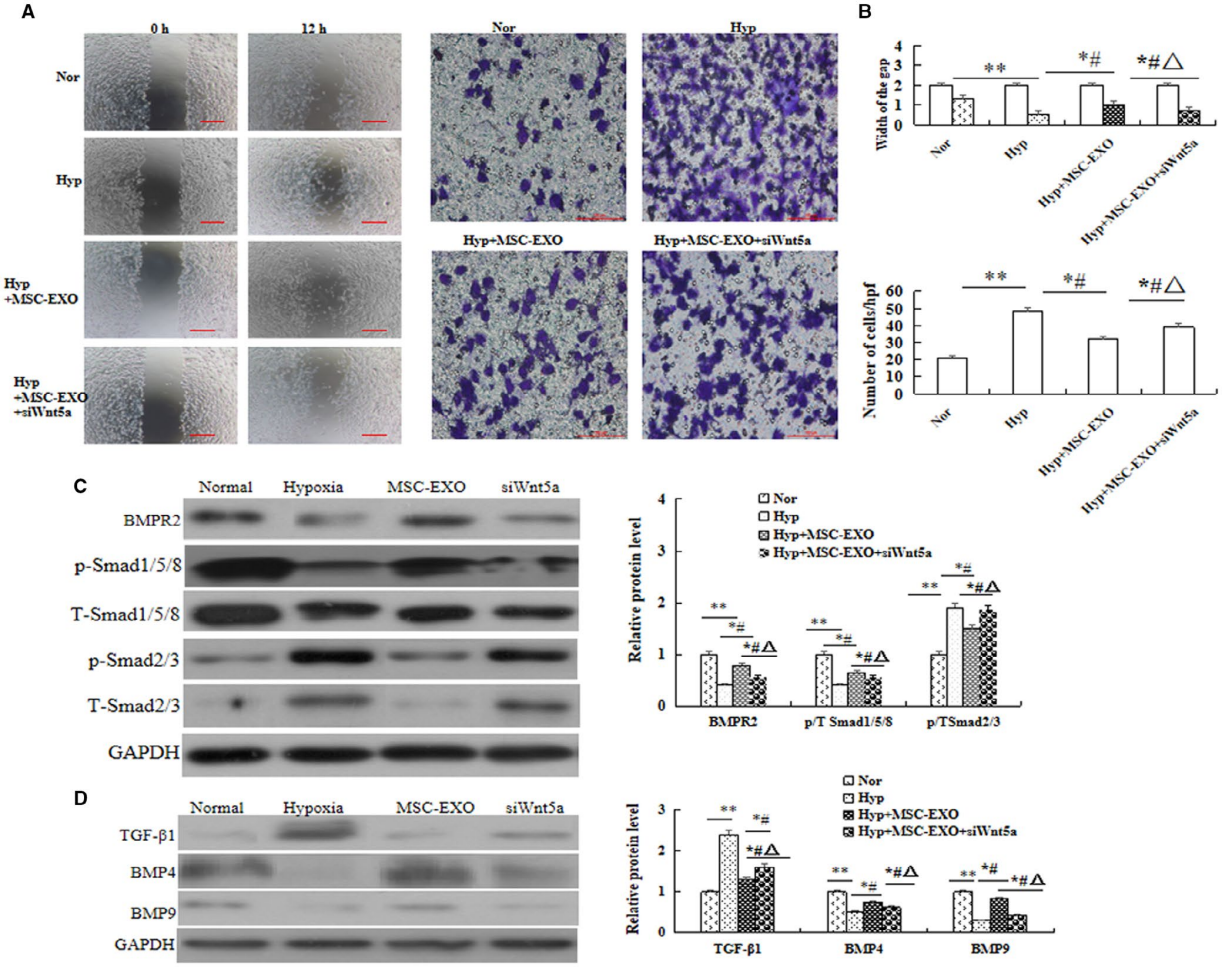
Effect of MSC‐EXO on PAEC invasion, migration and protein expression. A, Wound healing assay and Transwell assay in PAEC. B, Comparative analysis of invasion and migration ability. C, Protein expression and comparative of BMPR2, P/T‐Smad1/5/8 and P/T‐Smad2/3. D, Protein expression of TGF‐β1, BMP4 and BMP9. *P < 0.05 vs Normal group; ^#^ *P* < 0.05 vs hypoxia group; ^△^ *P* < 0.05 vs MSC‐EXO group. Red bar = 100 µm

And then the proliferation of PAECs and PASMC was measured by CCK8 and BrdU Cell Proliferation Assay Kit, and the results indicated that the viability and proliferation of PAEC at hypoxic exposure 48 hour and the proliferation of PASMC at 24h, 48h and 72h were significantly reduced in MSC‐EXO group as compared with hypoxic exposure (Figure 4A‐C *P* < 0.05). On the other hand, the protein expression of PCNA and Ki67 in PAEC was also detected by Western blot and immunofluorescence. The results showed that the protein expression of PCNA and Ki67 were obviously decreased in PAEC with MSC‐EXO treatment group as compared with hypoxia group (Figure 4D,E *P* < 0.05). We also analysed the invasion and migration ability of the hypoxia‐PAECs by wound healing and Transwell assays, and the results showed MSC‐EXO could significantly inhibit hypoxia‐PAEC invasion and migration (*P* < 0.05, Figure 5A,B). More importantly, the above results were significantly reversed in Wnt5a siRNA group (*P* < 0.05).

### 3.5 Effect of MSC‐EXO on BMP signalling pathway in hypoxia‐induced PAEC

**Figure 6:**
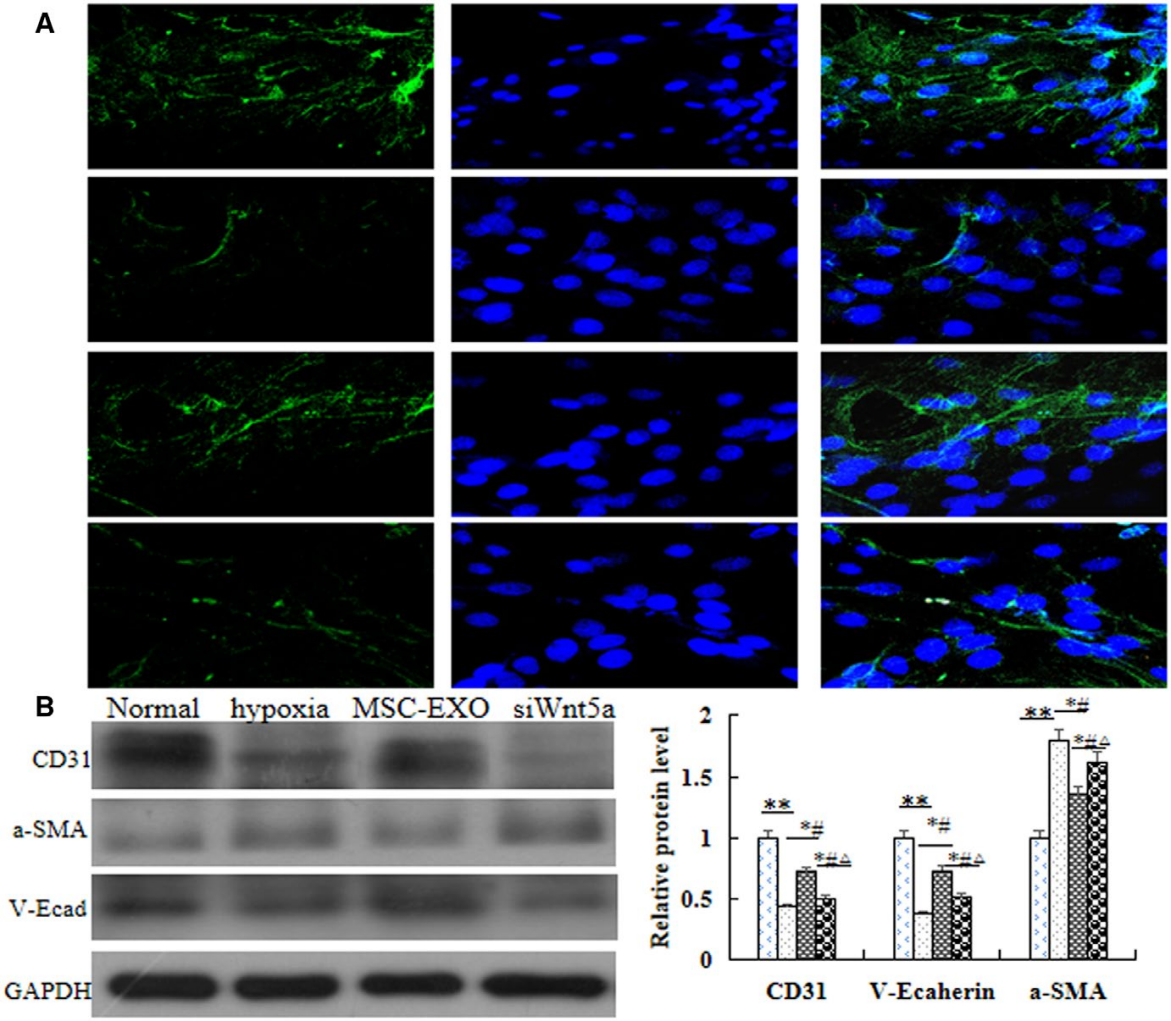
Effect of MSC‐EXO on hypoxia‐induced EndMT in PAECs. A, Immunofluorescence analysis of a‐SMA and V‐E cadherin. B, Protein expression and comparative analysis of CD31, a‐SMA and V‐E cadherin in PAEC. *P < 0.05 vs Normal group; ^#^ *P* < 0.05 vs hypoxia group; ^△^ *P* < 0.05 vs MSC‐EXO group. Red bar = 100 µm

To evaluate the interaction between BMP and Wnt signalling in vitro, the protein expression of BMP signalling‐related molecules were analysed. Western blot results indicated that the protein expression of BMPR2, the ratio of phosphorylated (P) to total Smad1/5/8, TGFβ1, BMP4 and BMP9 were significantly increased, but TGF‐β1 and the ratio of P to total Smad 2/3 were significantly decreased in MSC‐EXO group as compared with hypoxia group (*P* < 0.05). Moreover, these results were significantly reversed when the cells were transfected with Wnt5a siRNA (*P* < 0.05, Figure 5C,D). In order to observe the effect on hypoxic‐induced cell adhesion and contraction, the protein expression of endothelial cell marker CD31, smooth muscle cell markers a‐SMA and adherens junction protein of V‐E cadherin was measured by immunofluorescence and Western blot. The results showed that the levels of CD31 and V‐E cadherin were significantly higher, α‐SMA was significant lower in MSC‐EXO group than that in hypoxia group (*P* < 0.05), and these results were also be reversed after cells were transfected with Wnt5a siRNA. (*P* < 0.05, Figure 6A,B). Collectively, the above data suggested that the inhibition effect of MSC‐EXO on EndMT and promotion adhesion ability is associated with regulation Wnt5a/BMPR2 signalling pathway.

## 4 DISCUSSIONS

Our present study demonstrated that administration of MSC‐EXO could significantly reduce pulmonary hypertension (PH) vascular remodelling, through regulation of Wnt5a/BMP signalling pathway. The experiment was performed with unbiased approaches.^12^

Wnts are a family of secreted glycoproteins with varying expression patterns and a range of functions, and Wnt signalling pathway is divided into canonical signalling pathway and non‐canonical signalling pathway. Previous research suggests that Wnt signalling can involve in the pathogenesis of PH.^15^, ^16^ Canonical and non‐canonical Wnt pathways can promote angiogenesis and inhibit vascular regression in idiopathic PH. Wnt5a‐mediated non‐canonical signalling has been shown to regulate endothelial cell proliferation and migration and inhibit hypoxia‐induced PASMC proliferation via suppression of β1‐catenin/cyclin D1.^1^, ^10^, ^17^, ^18^ Endothelial cell‐derived non‐canonical Wnt ligands as regulators of Endothelial cell survival, proliferation and subsequent vascular pruning during developmental and pathological angiogenesis. Loss of Wnt5a derived from endothelia will let endothelial cell polarization direction against the blood flow direction in the low wall shear stress.^1^, ^10^ The results of this study showed that the expression of Wnt5a was significantly increased; β‐catenin and cyclin D1 were decreased in MSC‐EXO group as compared with MCT or hypoxia group in vivo or vitro. However, when WNT5a gene silence, the inhibition of MSC‐EXO on hypoxia‐induced PAEC or PAMSC proliferation was significantly reduced, these results confirmed that the mechanism of MSC‐EXO on PH pulmonary vascular remodelling was through regulation of Wnt5a/β‐catenin.

Bone morphogenetic proteins (BMPs) and their receptors were the key factors for PH vascular remodelling, which could regulate cell proliferation, migration, differentiation and apoptosis.^19^, ^20^ Studies showed more than 70% of heritable cases of PH and approximately 20% of apparently.^2^, ^21^ The balance between TGF‐β1 and BMP signalling plays a crucial role in pulmonary fibrosis.^22^ BMPs stimulate activation of TGF‐β1/SMAD transcription factors through heteromeric receptor complexes.^23^, ^24^ Reduced BMPR2 expression can impair PAEC function including influence apoptosis and tube formation and promote PASMC proliferation.^25^, ^26^ BMP signaling can induce tandem recruitment of the Wnt/β‐catenin pathway to induce motility and suppress proliferation of vascular smooth muscle cell, thereby regulating their contribution to vascular remodeling after vascular injury.^19^, ^27^, ^28^ Therefore, the effect of MSC‐EXO regulation of Wnt5a/BMP signalling against PH needs further research. In our study, we silence WNT5a gene in hypoxia‐PAEC to reveal the mechanism of MSC‐EXO. Our results showed that the expression of BMPR2, BMP4, BMP9 and p‐Smad1/5/8 related to the total Smad levels were significantly increased, but TGF‐β1 and p‐Smad2/3 related to the total Smad 2/3 were significantly decreased in MSC‐EXO group than that in hypoxia groups, however, which were significantly reversed in WNT5a gene silence group. These results suggested that the underlying mechanism of MSC‐EXO on PH pulmonary vascular remodelling was through regulation of Wnt5a/BMP signalling pathway, further to down‐regulation of phospho‐Smad2/3 and up‐regulation of phospho‐Smad1/5/8.

A large number of studies have shown that there was the potential role of EndMT in vascular remodelling and the fibrotic lung disease.^29^, ^30^ Endothelial cells undergoing EndMT lose their surface marker protein, such as CD31 and vascular endothelial cadherin (V‐E cadherin), but acquire mesenchymal phenotype α‐SMA. Reports showed that EndMT partially increased was associated with lower BMPR2 expression.^20^, ^21^ Our histology, immunofluorescence and Western blot results showed that all the cells in the lung tissue were proliferation, including PASMC, PAEC and fibroblast, respectively. However, the expression of CD31 and V‐E cadherin were significant lower, but α‐SMA was higher in PH and hypoxia groups. These indicated that the proliferation of PAEC was mainly the process of EndMT. More importantly, our present study showed that CD31 and V‐E cadherin were significantly increased, but α‐SMA was significantly decreased in MSC‐EXO group as compared with PH or hypoxia groups. These suggested that MSC‐EXO reduced PH vascular remodelling was through inhibition of EndMT, which maybe related with regulation of BMPR2 signalling pathway. On the other hand, non‐canonical Wnt signalling through regulation of cell migration and cell polarity to control vascular remodels.^31^ Wnt5a helps cells to move together by stabilizing vinculin at cell junctions and play an important role in endothelial cell migration.^18^ VE‐cadherin is the major transmembrane component of endothelial adherens junctions and a major effector of cell polarity,^32^, ^33^, ^34^, ^35^ levels of β‐catenin can be regulated through the canonical Wnt signaling or through the intracellular adhesion complex where it binds to VE‐cadherin, regulation of EC survival, proliferation and subsequent vascular pruning during developmental and pathological angiogenesis.^36^, ^37^, ^38^ Furthermore, our results showed that siRNAWnt5a transfection could reverse the expression of V‐E cadherin. Taken together, in the present study, our results confirmed that MSC‐EXO could significantly suppress the vascular remodelling and right ventricular hypertrophy induced by MCT, which through regulation of Wnt5a and/or BMPR2 signalling pathway and then inhibition of EndMT process. However, the mechanisms remain to be elucidated. A lot of studies^39^, ^40^, ^41^ suggested that EXO is the effective vehicle for the intracellular delivery of microRNAs (miRs). Recent studies proposed that miRs are the key factors in regulation of pulmonary vascular remodelling and may provide new therapeutic targets for PH.^42^ Dysregulation of miRs can target regulation of major protein factors including Wnt/β‐catenin^43^ and BMPR2^44^ in PH. Collectively, the results of the present study suggest further study of the regulation mechanism of miRs on Wnt5a/BMRR2 signalling pathway will be very valuable.

### 4.1 Limitations

MCT animal models do not reproduce the full spectrum of changes seen in lung specimens from idiopathic pulmonary arterial hypertension patients, and it also involves the liver and an inflammatory response. However, it is also very valuable to validate new targets and/or treatments and to give insights into the disease mechanisms.^40^ While Sugen‐Hypoxia rats display plexiform lesion in pulmonary artery in the lung, a key feature of human PH, MCT rats develop right ventricle failure, consistent with patients with PH with right ventricle decompensation.^14^ Among preclinical models of PH, MCT animal models offer the advantage of being able to mimic several key aspects of human PH, including vascular remodelling, proliferation of smooth muscle cells, endothelial dysfunction and right ventricle failure, which requires only a single drug injection.^39^, ^41^ The fact is that no single animal model can capture all the features of human PH. Nevertheless, studying the effect of MSC‐EXO on vascular remodelling and right ventricular hypertrophy from Sugen‐Hypoxia rats and PH patients would be an important future direction of study. On the other hand, previous studies have showed that the transplanted exosomes, not only in the brain tissue but also in the peripheral organs such as the lung, liver and spleen, here, verify the MSC‐EXO to the lungs in vivo experiments is missing, those cannot be clarified in the present study and would need further investigations.

## 5 CONCLUSION

In summary, the present study showed for the first time that MSC‐EXO increased the expression of Wnt5a further to regulate BMPR2 signalling pathway to prevention and treatment of PH vascular remodelling. Those cannot be clarified in the present study and would need further investigations.

## CONFLICT OF INTEREST

All authors declare no conflict of interest.

## AUTHOR CONTRIBUTIONS

**Zhaohua zhang:** Conceptualization (supporting); Data curation (supporting); Formal analysis (lead); Investigation (lead); Methodology (supporting); Project administration (supporting). **Lili Ge:** Conceptualization (lead); Data curation (lead); Investigation (lead). **Shanshan zhang:** Data curation (lead); Formal analysis (supporting); Investigation (supporting).

## Supporting information

Fig S1
