# Supplementary figures and images for: The protective effects of MSC‐EXO against pulmonary hypertension through regulating Wnt5a/BMP signalling pathway
Source: J Cell Mol Med. 2020 Oct 22;24(23):13938–48. doi: 10.1111/jcmm.16002 (PMC7754064; doi:10.1111/jcmm.16002)

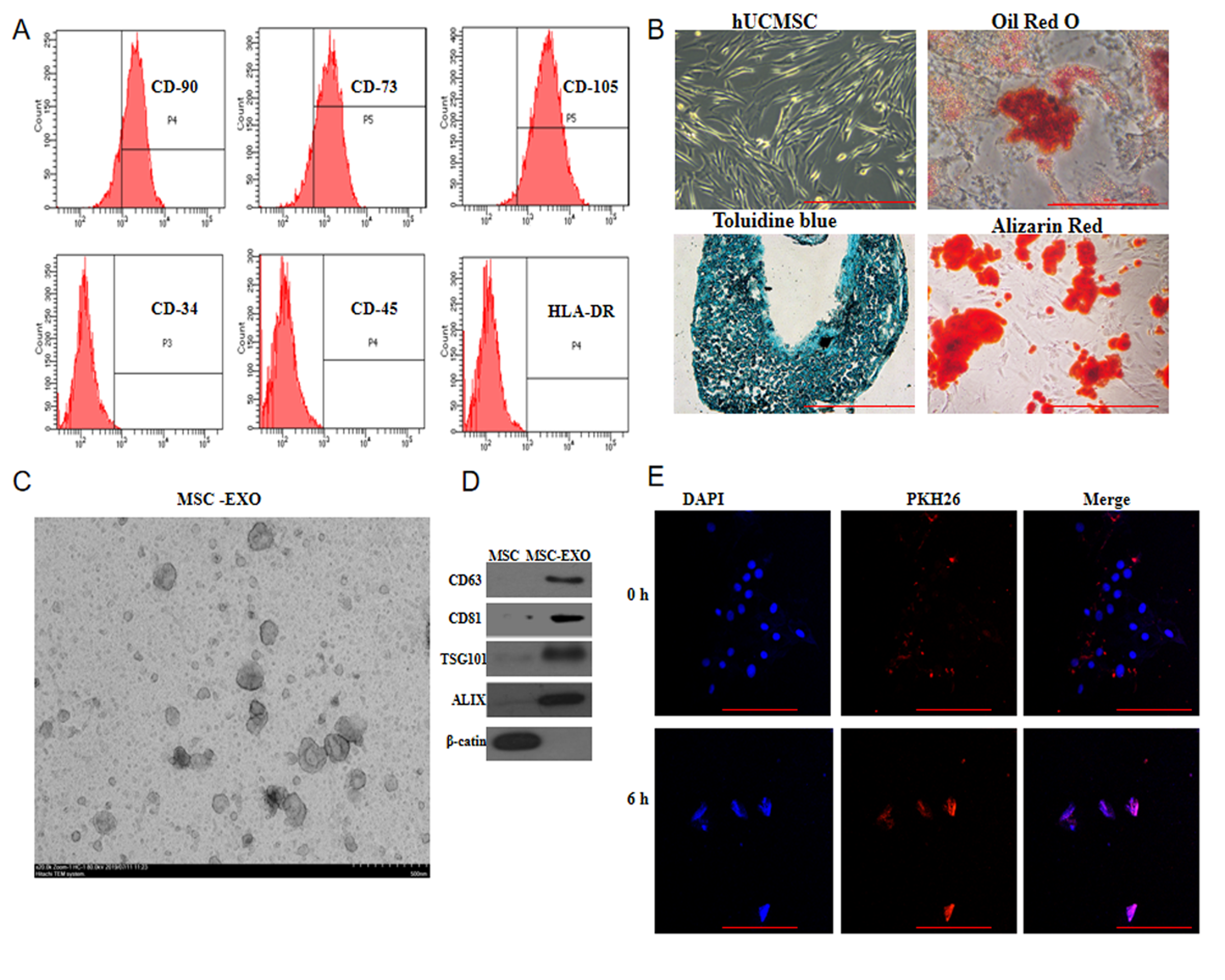

Supplement: Supplementary file 1 — Fig S1 [file JCMM-24-13938-s001.tif]
